# Prediction for HBsAg seroconversion in children with chronic hepatitis B

**DOI:** 10.1186/s12879-021-06883-1

**Published:** 2021-12-04

**Authors:** Yan-Wei Zhong, Yan-Min Shi, Fang Chu, Jie Liu, Ce Shi, Jiao-Jiao Xu, Peng Liu, Yan-Jie Bai, Xiao-He Xiao, Xiu-Chang Zhang, Min Zhang

**Affiliations:** 1 Senior Department of Hepatology, the Fifth Medical Center of Chinese PLA General Hospital, Xisihuan Mid-Road No.100, 100039 Beijing, China; 2 Hebei North University, South Diamond Road No.11, High Tech Zone, Zhangjiakou Province, 075000 Hebei China; 3 Peking University Third Hospital, 49 North Garden Rd., Beijing, 100191 China

**Keywords:** Children, Chronic hepatitis B, HBsAg seroconversion, HBV cccDNA, HBsAg

## Abstract

**Background:**

To establish a prediction of HBsAg seroconversion in children with chronic hepatitis B (CHB), so as to help clinicians to choose therapeutic strategy.

**Methods:**

A total of 63 children with HBeAg-positive CHB aged 1 to 17 years, who admitted to the fifth medical center of Chinese PLA general hospital and treated with interferon α (IFNα) 48 weeks were enrolled, the clinical data were measured. Based on the results of HBsAg seroconversion (HBsAg < 0.05 IU/mL and anti-HBsAg > 10 IU/L) at week 48, the patients were divided into HBsAg seroconversion (S) group and non-HBsAg seroconversion (NS) group. Multivariate COX regression was used to identify the impact factors associated with HBsAg seroconversion. A novel prediction index was established and the area under the receiver operating characteristic curve (AUROC) was used to assess the prediction for HBsAg seroconversion.

**Results:**

The 63 patients were divided into S group (20.6%, 13/63) and NS group (79.4%, 50/63). Univariate and multivariate analysis identified age, baseline intrahepatic cccDNA and serum HBsAg levels were independent impact factors for HBsAg seroconversion. Intrahepatic cccDNA was positively correlated with serum HBsAg (r = 0.464, *p* = 0.000). AUROC of HBV cccDNA was 0.83 (95% CI 0.71 to 0.95) and AUROC of baseline HBsAg was 0.77 (95% CI 0.61 to 0.92). Intrahepatic cccDNA ≤ 0.08 log_10_ copies/10^6^ cell is regarded as cutoff value, the positive predictive value(PPV) and negative predictive value(NPV) for HBsAg seroconversion were 86.8% and 60.0%, respectively, with a sensitivity of 92.0% and specificity of 56.2%. HBsAg ≤ 3.68 log_10_ IU/mL is used as cut off value, the PPV and NPV for HBsAg seroconversion were 91.2% and 56.3%, respectively; the sensitivity and specificity was 86.0% of 69.2%, respectively. There was no statistical difference between them for predicting HBsAg seroconversion (*p* = 0.146).

**Conclusions:**

HBsAg seroconversion can be predicted by the baseline serum HBsAg or intrahepatic cccDNA in children with CHB. Using the index, clinicians can choose more reasonable therapeutic strategy and reduce the waste of medical resources.

## Background

The patients who acquired hepatitis B virus (HBV) infection in childhood have an enhanced risk for the chronic hepatitis B (CHB), cirrhosis and liver cancer in adulthood [1]. HBsAg loss or seroconversion is the ideal endpoint of antiviral therapy. Effective antiviral treatment is the key to improve the rates of HBsAg loss or seroconversion in patients with CHB [2]. The HBV covalently closed circular DNA (cccDNA) is the original template for HBV DNA replication and the basis of HBV persistent infection, which has been used as an important indicator of evaluating antiviral effect in patients with CHB [3, 4]. As alternative index of HBV cccDNA, hepatitis B surface (HBsAg) has been used as novel serum markers for prediction of prognosis and treatment response in adults [5–7]. However, up until now, the predictive value of HBV cccDNA and serum HBsAg for HBsAg seroconversion in children with CHB remains unknown. Therefore, establishing a prediction index of HBsAg seroconversion become desirable. In this study, we developed a HBsAg seroconversion predictive index of children with CHB can help clinicians to better choose therapeutic strategy. To our knowledge, this is the first prediction index for HBsAg seroconversion in children with CHB.

## Methods

### Study design

A total of 63 children with HBeAg-positive CHB, aged 1–17 years were enrolled, who treated with IFN between February 2016 and February 2019. Based on the results of HBsAg seroconversion (HBsAg < 0.05 IU/mL and anti-HBs > 10 IU/L) at 48 weeks, the patients were divided into HBsAg seroconversion(S) group and non-HBsAg seroconversion (NS) group. Age, sex, baseline HBV DNA, ALT, HBV cccDNA, HBsAg levels, genotype, degree of hepatic inflammation and fibrosis were measured. Univariate and multivariate COX regression analysis was used to identify the impact factors associated with HBsAg seroconversion. A novel prediction index was established. The area under the receiver operating characteristic curve(AUROC) were used to evaluate the predictive value for HBsAg seroconversion of baseline intrahepatic cccDNA and HBsAg in children with CHB receiving 48 weeks antiviral therapy.

### Study population

This study was approved by the fifth medical center of Chinese PLA general hospital, Beijing, written informed consent was waived by patients` parents. Sixty-three pediatric inpatients with HBeAg positive CHB who visited Beijing 302 Hospital from February 2016 to February 2019 were enrolled in the study. All patients were anti-HCV and anti-HIV negative (34 males and 29 females, mean age 5.48 ± 2.64 years; range 0.97–17 years. The main characteristics were patients with persistent elevated ALT levels and high HBV load). The diagnostic and treatment criteria were based on “2012 European Association for the Study of the Liver” and Management Scheme of Diagnostic and Therapy Criteria of Viral Hepatitis, issued by the Chinese Society of Infectious Diseases and Parasitology and the Chinese Society of Hepatology, which have been described in detail in our previous studies [8, 9].

Patients with CHB enrolled in this study met the criteria listed as follows: (1) hepatitis B surface antigen (HBsAg) positive serum of no less than 6 months from the initial onset; (2) serum HBV DNA level more than 10^5^ copies/mL; (3) continuous or interval rise of alanine transaminase (ALT)/aspartate transaminase (AST) level; (4) HBeAg positive, anti-HBe negative. The exclusion criteria: (1) previous antiviral treatment for HBV infection; (2) there was no evidence for hepatocellular carcinoma (HCC), or concomitant of HCV, HDV, HIV coinfection and autoimmune liver disease, drug-induced liver injury or Wilson’s disease, liver transplantation. 63 HBV-infected children with persistent elevated ALT levels and high HBV load without jaundice and no evidence of other comorbidities were carried out recombinant human interferon alpha(IFNα), subcutaneous injection (3 MU/m^2^ to 5 MU/m^2^, qod) and undergone liver biopsy.

### Serological markers and quantification of HBV DNA, HBV cccDNA and HBsAg levels

Blood was collected on the day of the liver biopsy. Serum ALT, HBeAg/anti-HBe and other serological markers were routinely measured in the central clinical laboratory of the fifth medical center of Chinese PLA general hospital. Serum HBV DNA levels were determined by real-time quantitative PCR kit (Fosun Pharmaceutical Co., Ltd., Shanghai, China) with a lower detection limit of 100 IU/mL (about 500 copies/mL). Serum HBsAg levels were quantified by Electro-Chemiluminescence Immunoassay using the Roche COBAS 8000 (Roche diagnostics co, Switzerland), according to the manufacturer’s instructions, HBsAg > 0.05 IU/mL was considered to be positive results. Intrahepatic HBV cccDNA levels were measured by plasmid-safe ATP dependent Danes (PSAD) digestion in combination with rolling circle amplification (RCA) and gap-spanning selective real-time PCR assay as described previously [10]. Intrahepatic HBV cccDNA levels were normalized by the amount of human genomic (hg)-beta actin in the samples. Cell numbers were calculated based on an estimation of 6.667 pg/hgDNA per cell. HBV genotype was analyzed as described previously [11]. The stage of fibrosis and the degree of inflammatory activity were evaluated by the Metavir score system [12].

### Clinical monitoring

Patients were monitored every 3 months, including blood routine test, thyroid function, autoantibody test, HBsAg quantification, body temperature and mental state.

### Statistical methods

When the data present normality or non normality, continuous variables were displayed as means ± standard deviation (SD) or median (interquartile range). The differences between normal data groups were compared using t-test (homogeneity of variance) or adjusted t-test (heterogeneity of variance), and non normal data were analyzed using Wilcoxon test. The calculation of the frequency or proportion of those classified variables for each type of patients was performed, and the differences between the groups was analyzed using x^2^ test. All data were measured bilaterally, and p value was statistically significant under 0.05 [13]. The univariate and multivariate analysis was performed for investigating the independent impact factors of HBsAg seroconversion. Area under the receiver operating characteristic (ROC) curve analysis was performed to predict the likelihood of achieving HBsAg seroconversion.

## Results

### Clinical characteristics of patients

The baseline characteristics of the patients were shown in Table 1. As the Table 1 showed that a total of 63 cases were enrolled, including 34 males and 29 females, aged 0.97–17 years, mean age was 5.48 ± 2.64 years. Among of them, 20.6% (13/63) patients achieved HBsAg seroconversion at 48 weeks, 34.9% (22/63) achieved HBeAg seroconversion at the week 48, 26.98% (17/63) patients achieved HBsAg seroconversion and 41.27% (26/63) patients experienced HBeAg seroconversion at post-treatment 6 months, all of them had not relapse within 2 years after the end of treatment. Baseline HBV DNA levels were 7.39 ± 1.45 log_10_ IU/mL, HBsAg levels were 4.01 ± 0.86 log_10_ IU/mL, HBV cccDNA levels were 6.64 ± 0.74 log_10_ copies/10^6^cell, ALT levels were 95.00 (57.00, 170.00). Because ALT values presents a skew distribution, we used the median and quartile to describe it. 16 patients (25.4%) out of the total 63 were infected with genotype B, whereas the rest patients (74.6%) were infected with genotype C. Based on the results of HBsAg seroconversion (HBsAg < 0.05 IU/mL and anti-HBs > 10 IU/L) at 48 weeks antiviral treatment, the patients were divided into HBsAg seroconversion(S) group and non-HBsAg seroconversion (NS) group. There were significant statistical differences between the S and NS group with respect to the age *(p* = 0.000), cccDNA levels (*p* = 0.040), HBsAg levels (*p* = 0.009) at baseline, however, there were no statistical differences were observed regarding the gender (*p* = 0.208), ALT levels(*p* = 0.108), HBV DNA (p = 0.063), genotype (*p* = 1.000), degree of hepatic inflammation (*p* = 0.082) and fibrosis stage(*p* = 0.663) at baseline between both groups.

**Table 1:** Baseline characteristics of the pediatric patients with HBeAg positive CHB

Variable	Overall (n = 63)	HBsAg seroconversion(n = 13)	Non-HBsAg seroconversion(n = 50)	*P-value*
Age(years)	5.48 ± 2.64	1.68 ± 0.71	6.47 ± 3.16	0.000
ALT > 2 × ULN, n(%)	39(61.9)	7(53.8)	32(64.0)	0.108
Serum HBV DNA (log_10_ IU/mL)	7.39 ± 1.45	6.72 ± 1.52	7.56 ± 1.34	0.063
HBV cccDNA, (log_10_ copies/10^6^ cell)	6.64 ± 0.74	6.38 ± 0.79	6.79 ± 0.66	0.040
Serum HBsAg (log_10_ IU/mL)	4.01 ± 0.86	3.48 ± 1.22	4.14 ± 0.69	0.009
Gender (n, %)				0.208
Male	34(54.0%)	5(14.7%)	29(85.3%)	
Female	29(46.0%)	8(27.6%)	21(72.4%)	
HBV genotype (n, %)				1.000
B	16(25.4%)	3(18.7%)	13(81.3%)	
C	47(74.6%)	10(21.3%)	37(78.7%)	
Grade of inflammation (n, %)				0.082
G0–G1	30(47.6%)	9(30.0%)	21(70.0%)	
≥ G2	33(52.4%)	4(12.1%)	29(87.9%)	
Histological fibrosis stage (n, %)				0.663
S0–S1	48(76.2%)	11(22.9%)	37(77.1%)	
≥ S2	15(23.8%)	2(13.3%)	13(86.7%)	

*CHB* chronic hepatitis B; *HBsAg* hepatitis B surface antigen; *ALT* alanine transaminase, the upper limit of normal for ALT is ALT < 1 × ULN; *HBV cccDNA* hepatitis B virus covalently closed circular DNA

### Independent impact factors associated with HBsAg seroconversion

Further multivariate COX analysis showed that the intrahepatic HBV cccDNA levels (HR 0.173, 95% confidence interval (CI) 0.036–0.820, p = 0.047); serum HBsAg levels (HR 0.217, 95% CI 0.047–1.006, p = 0.049) and age (HR 0.318, 95% CI 0.129–0.784, p = 0.013) were independent influence factors associated with HBsAg seroconversion for those patients who received 48 weeks antiviral therapy (Table 2). Interestingly, there was a high rate of HBsAg seroconversion among the patients under 5 years old compared to older 5 years (69.2% vs 30.8%, p < 0.05).

**Table 2:** Multivariate COX analysis of HBsAg seroconversion

Factors	B	S.E	Wald	P	HR	95% CI
HBV cccDNA in liver tissues	− 1.757	0.795	4.885	0.047	0.173	0.036–0.820
Serum HBsAg	− 1.528	0.783	3.811	0.049	0.217	0.047–1.006
Age	− 1.145	0.460	6.199	0.013	0.318	0.129–0.784

*HBsAg* hepatitis B surface antigen; *HBV cccDNA* hepatitis B virus covalently closed circular DNA

### The correlations analysis

**Fig. 1:**
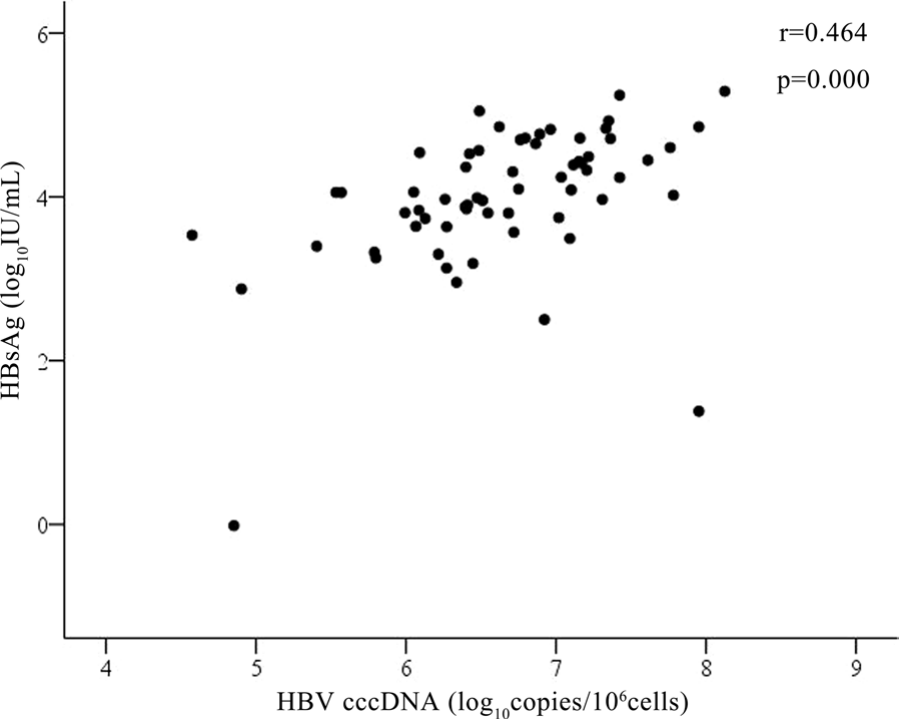
Correlation between intrahepatic hepatitis B virus covalently closed circular DNA (HBV cccDNA) level and baseline serum hepatitis B surface antigen (HBsAg) level

The relationship between baseline HBV cccDNA and serum HBsAg levels was showed in Fig. 1. The results revealed that baseline intrahepatic cccDNA levels was positively correlated with serum HBsAg levels (r = 0.464, *p* = 0.000).

**Fig. 2:**
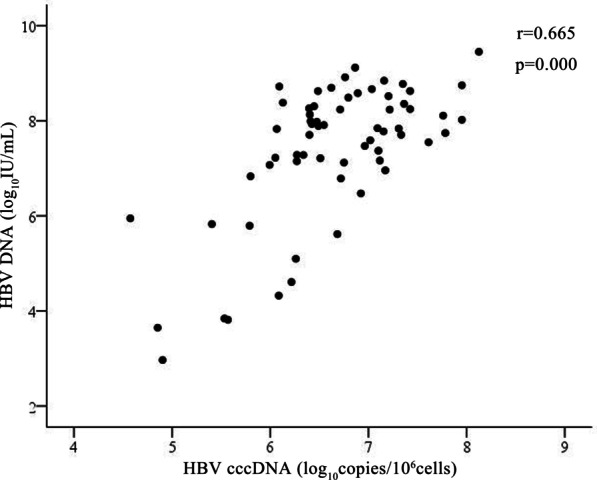
Correlation between intrahepatic hepatitis B virus covalently closed circular DNA (HBV cccDNA) level and baseline hepatitis B virus (HBV) DNA level

Furthermore, Fig. 2 showed HBV cccDNA levels was also positively correlated with HBV DNA levels (r = 0.665, *p* = 0.000).

**Fig. 3:**
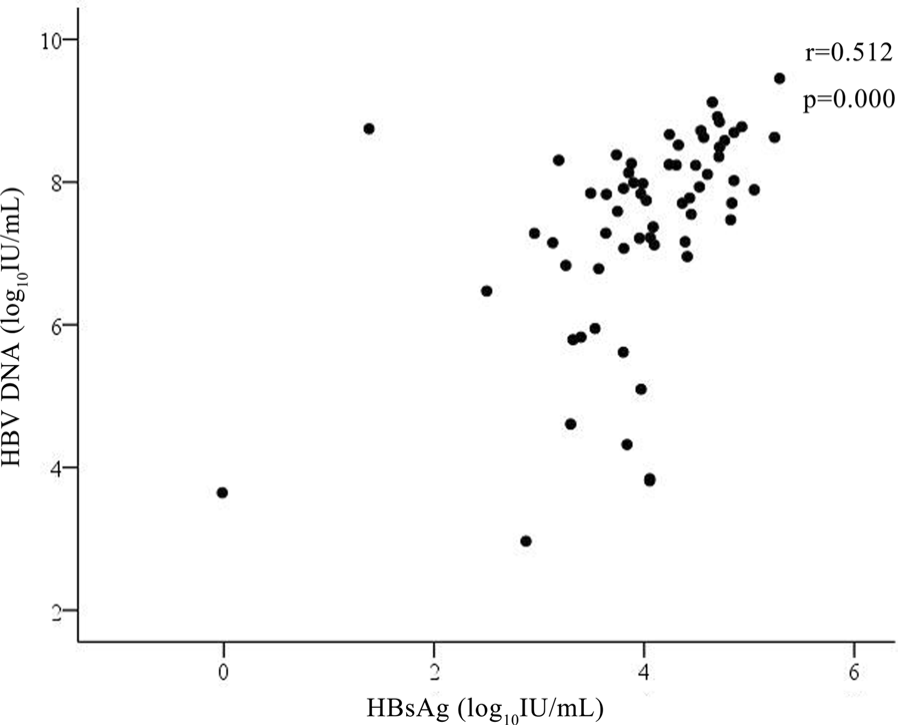
Correlation between baseline serum hepatitis B surface antigen (HBsAg) level and baseline hepatitis B virus (HBV) DNA level

As demonstrated in Fig. 3, there was positively correlated between serum HBsAg and HBV DNA levels at baseline (r = 0.512, *p* = 0.000). However, there was no correlation was observed between ALT and cccDNA levels (r = 0.251, *p* = 0.057), as well as HBsAg levels (r = 0.106, *p* = 0.407) at baseline.

### Construction and assessment of a novel predict index

In order to facilitate clinical use and further assessment, a novel predict index was established. To evaluate the role of HBV cccDNA / HBsAg in predicting HBsAg seroconversion in patients with CHB at week 48, areas under the receiver operating characteristic (AUROC) was performed to predict the HBsAg seroconversion in HBeAg-positive children who received 48 weeks antiviral treatment.

**Fig. 4:**
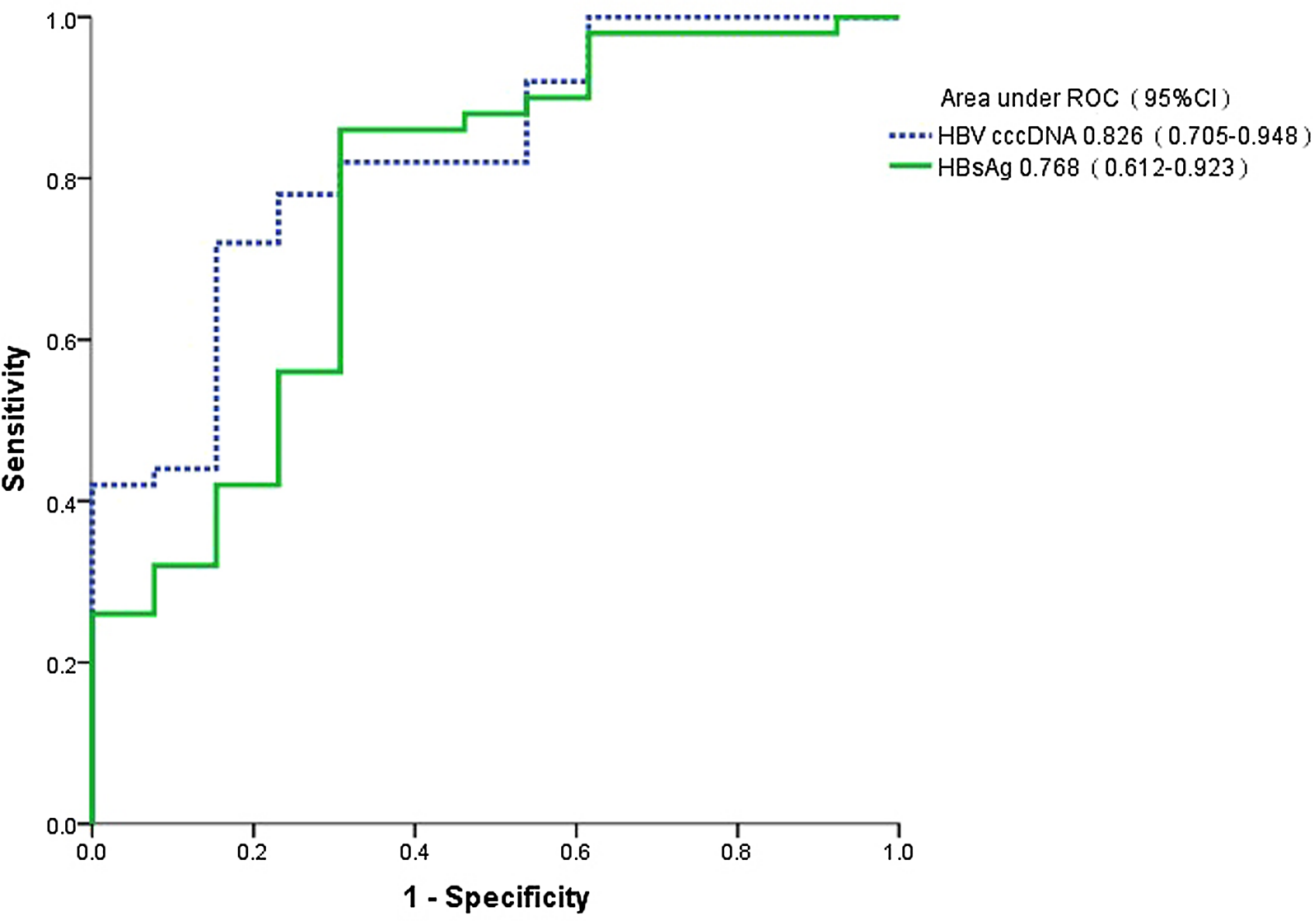
Receiver operating characteristic (ROC) curve of hepatitis B virus covalently closed circular DNA (HBV cccDNA) and serum hepatitis B surface antigen (HBsAg) levels for predicting HBsAg seroconversion in pediatric patients with chronic hepatitis B (CHB) receiving antiviral treatment 48 weeks

As illustrated in Fig. 4, AUROC of intrahepatic HBV cccDNA and serum HBsAg level was 0.826 (95% CI 0.71–0.95) and 0.768 (95% CI 0.61–0.92), respectively. According to maximum the youden index, the point with the largest area under the ROC curve is the optimal diagnostic point, which is used as the cut off value of cccDNA and HBsAg, respectively. Using intrahepatic cccDNA ≤ 0.08 log_10_ copies/10^6^ cell as cutoff value, the positive predictive value (PPV) and negative predictive value (NPV) for HBsAg seroconversion were 86.8% and 60.0%, respectively, with a sensitivity of 92.0% and specificity of 56.2%. Using HBsAg ≤ 3.68 log_10_ IU/mL as cut off, the PPV and NPV for HBsAg seroconversion were 91.2% and 56.3%, respectively, with a sensitivity of 86.0% and specificity of 69.2%.

### Comparation of the difference between HBV cccDNA and HBsAg for the predicting HBsAg seroconversion

We used paired chi-square test to compared the difference of AUROCs between intrahepatic cccDNA and serum HBsAg for predicting HBsAg seroconversion. There was no significant statistical difference between two parameters in predicting the HBsAg seroconversion for those patients who received 48 weeks antiviral treatment (*p* = 0.146). These data indicated that HBsAg and cccDNA levels had same important role in predicting HBsAg seroconversion of pediatric patients with CHB.

### Safety monitoring

Long-term follow-up results showed that early initiation of antiviral therapy with IFN may led to a rapid and significant serum HBsAg loss. Fever was the most common adverse event, in addition to this issue, no serious adverse events were observed in present study.

## Discussion

Antiviral therapy in children with CHB is one of the most controversial clinical problems. Most of children infected with HBV during perinatal period are usually asymptomatic and have normal alanine aminotransferase (ALT) levels. The guidelines do not recommend treatment. However, some patients may present hepatitis with continuously elevated alanine aminotransferase levels. Previous studies have shown that HBV infection in children under 6 years old is more likely occurred CHB. If patients are infected with HBV in infancy, the chronic rate of HBV infection will increase from 30 to 50% rising to 80% to 90% [14], which greatly increases the risk of adult cirrhosis and hepatocellular carcinoma. However, the current guidelines do not recommend reliable antiviral treatment for this population. In view of the current situation, in order to reduce the consequences of chronicity, experts suggest that active antiviral therapy should be considered to intervene the progress of HBV infection in children. IFN as a widely used antiviral drug had significant antiviral effect for patients with CHB [15]. Our team previously carried out an against HBV treatment for the infants with hepatitis B infection under 1 year old. We found that performing antiviral treatment early can make HBsAg clearance rapidly, moreover, most of them obtained HBsAg seroconversion, but the influence factors of HBsAg seroconversion in antiviral treatment are not clear. Therefore, we designed the present study to explore the predictors of HBsAg seroconversion in children with CHB.

We measured the levels of baseline HBsAg, intrahepatic HBV cccDNA, HBV DNA, ALT, genotype, degree of hepatic inflammation and fibrosis stage of pediatric patients with HBeAg positive CHB, and evaluated the association between the HBsAg seroconversion and above parameters. Our results showed that, compared with non-HBsAg seroconversion group, only age, baseline HBsAg and HBV cccDNA were significant difference between the two groups. Previous studies revealed that baseline higher ALT level and lower HBV DNA load was significantly associated with higher probability of HBsAg seroclearance [16–18]. However, in our study, there was no obvious correlation was observed among the ALT, HBV DNA load and HBsAg seroconversion, which were not consistent with the previous report in adults [16–18]. The immune system of pediatric patients developed incompletely and the immune response to infection might be different compared to that of adults might be the reasons for this difference [19]. Children were more susceptible to immunological tolerance than adults. Therefore, the ALT levels in children may not correctly reflect the degree of liver inflammation. HBV DNA was regarded as an important index of viral replication [16], but its expression levels were affected by many factors. Interestingly, in this study, there was a high rate of HBsAg seroconversion among the patients under 5 years old compared to older 5 years (69.2% vs 30.8%). The major reason may be shorter duration of HBV infection, which might be lead to higher HBsAg seroconversion rate. The previous studies had a similar finding about young age associated with higher HBsAg seroconversion rate after IFN treatment [15].

It has been suggested that achieving HBsAg seroclearance is the ideal endpoint of antiviral therapy [10–12, 14–22]. Effective antiviral therapy is the key to the treatment of CHB; however, some pediatric patients with CHB fail to obtain a virologic response after receiving antiviral therapy. One of the reasons leading to this result is its lack of reliable predictor. The previous study showed that antiviral efficacy was closely related to age, gender, HBV DNA, cccDNA levels, HBsAg, HBV genotypes, host immune status [23]. In the present study, multi-factor analysis showed that the levels of intrahepatic HBV cccDNA, the levels of serum HBsAg and the age of patients were closely related to the HBsAg seroconversion; the lower the baseline serum HBsAg, HBV cccDNA levels and the younger the patients, the better the antiviral treatment effect.

Some studies showed that the levels of serum HBsAg was closely related to the levels of intrahepatic cccDNA [5, 21], serum HBsAg could be considered as a surrogate marker to evaluate HBV cccDNA levels [5]. The results were supported by our study. Our data also revealed that the serum HBsAg levels at baseline was positively correlated with intrahepatic cccDNA levels in children with CHB. Previous reports showed that HBV DNA levels were correlated with HBsAg seroconversion. However, in our study, there was no significant correlation between baseline HBV DNA and HBsAg seroconversion. This might indicate that serum HBV DNA levels may not represent an ideal biomarker for evaluating intraheptic cccDNA production in the pediatric patients with CHB.

Ruan et al. [6] study showed that intrahepatic cccDNA was predictive for antiviral effect in CHB patients. Takkenberg et al. [18] reported that low baseline level of HBsAg was an effective factor to predict the antiviral effect of interferon in patients with HBeAg positive. However, the cutoff values of HBV cccDNA and HBsAg was still ambiguous. In order to obtain the predict data of HBsAg seroconversion in pediatric patients with CHB, we used the AUROC of HBsAg and HBV cccDNA levels to evaluate the predictive value for HBsAg seroconversion. From the ROC curves, we found that using intrahepatic cccDNA ≤ 0.08 log_10_ copies/10^6^ cell as cutoff, the PPV for HBsAg seroconversion was 86.8%; using HBsAg ≤ 3.68 log_10_ IU/mL as cut off, the PPV for HBsAg seroconversion was 91.2%. Although the AUROC of cccDNA was higher than that of HBsAg levels (0.826 vs 0.768), the difference was not statitically significant (p = 0.146). The detection of HBV cccDNA requires liver biopsy. The results of this study indicated that detection of HBV cccDNA can be replaced by serum HBsAg quantification.

The primary endpoint of this study was HBsAg seroconversion at 48 weeks of IFN alpha treatment. All predictors were analyzed according to this endpoint. However, it is conventional to determine the rates of HBeAg and HBsAg seroconversion at 6 or 12 months off IFN alpha treatment rather than at the end of treatment, which is a limitation of the present study.

## Conclusions

HBsAg seroconversion can be predicted through the baseline serum HBsAg levels. Baseline HBsAg ≤ 3.68 log_10_ IU/mL or intrahepatic cccDNA ≤ 0.08 log_10_ copies/10^6^ cell was a strong predicter for HBsAg seroconversion. Clinicians can use reasonably baseline HBsAg levels to perform individualized and optimized treatment for children with CHB.

## Data Availability

The datasets used and analyzed during the current study available from the corresponding author on reasonable request.

## Abbreviations

HBV: Hepatitis B virus
CHB: Chronic hepatitis B
HBeAg: Hepatitis Be antigen
HBsAg: Hepatitis B surface antigen
cccDNA: Covalently closed circular DNA
ALT: Alanine transaminase
AST: Aspartate transaminase
HCC: Hepatocellular carcinoma
PSAD: Plasmid-safe ATP dependent Danes
RCA: Rolling circle amplification
ROC: Receiver operating characteristic

## References

[1] Ligat G, Schuster C, Baumert TF (2019). Hepatitis B virus core variants, liver fibrosis, and hepatocellular carcinoma. Hepatology.

[2] Ko C, Chakraborty A, Chou WM, Hasreiter J, Wettengel JM, Stadler D (2018). Hepatitis B virus genome recycling and de novo secondary infection events maintain stable cccDNA levels. J Hepatol.

[3] Allweiss L, Volz T, Giersch K, Kah J, Raffa G, Petersen J (2018). Proliferation of primary human hepatocytes and prevention of hepatitis B virus reinfection efficiently deplete nuclear cccDNA in vivo. Gut.

[4] Bowden S, Locarnini S, Chang TT, Chao YC, Han KH, Gish R (2015). Covalently closed-circular hepatitis B virus DNA reduction with entecavir or lamivudine. World J Gastroenterol.

[5] Chuaypen N, Sriprapun M, Praianantathavorn K, Payungporn S, Wisedopas N, Poovorawan Y (2017). Kinetics of serum HBsAg and intrahepatic cccDNA during pegylated interferon therapy in patients with HBeAg-positive and HBeAg-negative chronic hepatitis B. J Med Virol.

[6] Ruan P, Zhou B, Dai X, Sun Z, Guo X, Huang J (2014). Predictive value of intrahepatic hepatitis B virus covalently closed circular DNA and total DNA in patients with acute hepatitis B and patients with chronic hepatitis B receiving anti-viral treatment. Mol Med Rep.

[7] Takkenberg B, Terpstra V, Zaaijer H, Weegink C, Dijkgraaf M, Jansen P (2011). Intrahepatic response markers in chronic hepatitis B patients treated with peginterferon alpha-2a and adefovir. J Gastroenterol Hepatol.

[8] European Association for the Study of the Liver (2012). EASL clinical practice guidelines: management of chronic hepatitis B virus infection. J Hepatol.

[9] Chinese Society of Hepatology, Chinese Medical Association; Chinese Society of Infectious Diseases, Chinese Medical Association, Hou JL, lai W. [The guideline of prevention and treatment for chronic hepatitis B: a 2015 update]. Zhonghua Gan Zang Bing Za Zhi. 2015;23(12):888–905. 10.3760/cma.j.issn.1007-3418.2015.12.002 PMC12677373 26739464

[10] Zhong Y, Han J, Zou Z, Liu S, Tang B, Ren X (2011). Quantitation of HBV covalently closed circular DNA in micro formalin fixed paraffin-embedded liver tissue using rolling circle amplification in combination with real-time PCR. Clin Chim Acta.

[11] Zhong YW, Li J, Song HB, Duan ZP, Dong Y, Xing XY (2011). Virologic and clinical characteristics of HBV genotypes/subgenotypes in 487 Chinese pediatric patients with CHB. BMC Infect Dis.

[12] Tonetto PA, Gonçales NS, Fais VC, Vigani AG, Gonçales ES, Feltrin A (2009). Hepatitis B virus: molecular genotypes and HBeAg serological status among HBV-infected patients in the southeast of Brazil. BMC Infect Dis.

[13] Wang JB, Wang ZX, Jing J, Zhao P, Dong JH, Zhou YF (2020). Exploring an integrative therapy for treating COVID-19: a randomized controlled trial. Chin J Integr Med.

[14] El-Raziky ME, Fouad HM, Abd Elkhalak NS, Ghobrial CM, El-Karaksy HM (2019). Paediatric chronic hepatitis B virus infection: are children too tolerant to treat?. Acta Paediatr.

[15] Zhu S, Dong Y, Wang L, Liu W, Zhao P (2019). Early initiation of antiviral therapy contributes to a rapid and significant loss of serum HBsAg in infantile-onset hepatitis B. J Hepatol.

[16] Su TH, Hsu CS, Chen CL, Liu CH, Huang YW, Tseng TC (2010). Serum hepatitis B surface antigen concentration correlates with HBV DNA level in patients with chronic hepatitis B. Antivir Ther.

[17] Kim GA, Lim YS, An J, Lee D, Shim JH, Kim KM (2014). HBsAg seroclearance after nucleoside analogue therapy in patients with chronic hepatitis B: clinical outcomes and durability. Gut.

[18] Takkenberg RB, Jansen L, de Niet A, Zaaijer HL, Weegink CJ, Terpstra V (2013). Baseline hepatitis B surface antigen (HBsAg) as predictor of sustained HBsAg loss in chronic hepatitis B patients treated with pegylated interferon-α2a and adefovir. Antivir Ther.

[19] Zhang Z, Chen D, Yao J, Zhang H, Jin L, Shi M (2007). Increased infiltration of intrahepatic DC subsets closely correlate with viral control and liver injury in immune active pediatric patients with chronic hepatitis B. Clin Immunol.

[20] Martinot-Peignoux M, Lapalus M, Asselah T, Marcellin P (2014). HBsAg quantification: useful for monitoring natural history and treatment outcome. Liver Int.

[21] Cornberg M, Wong VW, Locarnini S, Brunetto M, Janssen HLA, Chan HL (2017). The role of quantitative hepatitis B surface antigen revisited. J Hepatol.

[22] Li MH, Zhang L, Qu XJ, Lu Y, Shen G, Li ZZ (2017). The predictive value of baseline HBsAg level and early response for HBsAg loss in patients with HBeAg-positive chronic hepatitis B during pegylated interferon alpha-2a treatment. Biomed Environ Sci.

[23] Gao Y, Li Y, Meng Q, Zhang Z, Zhao P, Shang Q (2017). Serum hepatitis B virus DNA, RNA, and HBsAg: which correlated better with intrahepatic covalently closed circular DNA before and after nucleos(t)ide analogue treatment?. J Clin Microbiol.

